# Radioiodine (131I) treatment decision-making for low- and intermediate-risk differentiated thyroid cancer

**DOI:** 10.20945/2359-3997000000538

**Published:** 2023-01-17

**Authors:** Haiyan Gao, Jiyuan Huang, Qingjing Dai, Juan Su

**Affiliations:** 1 University of Electronic Science and Technology of China Sichuan Provincial People’s Hospital Department of Nuclear Medicine Sichuan Province China Department of Nuclear Medicine, Sichuan Provincial People’s Hospital, University of Electronic Science and Technology of China, Sichuan Province, China

**Keywords:** Differentiated thyroid cancer, radioactive iodine [^131^I] treatment, low and intermediate-risk

## Abstract

**Objective:**

The purpose of this study was to investigate the effect and influencing factors of post-surgical radioactive iodine (RAI) therapy for patients with low- and intermediate-risk differentiated thyroid cancer (DTC).

**Subjects and methods:**

A retrospective analysis of 423 low- and intermediate-risk DTC patients admitted to the Department of Nuclear Medicine, Sichuan Provincial People’s Hospital from January 2005 to December 2020 was performed. All patients were treated with surgery, had a postoperative pathological diagnosis, and were treated with RAI, including 89 males and 334 females. Recurrence risk stratification: 143 cases were low-risk, and 280 cases were intermediate-risk.

**Results:**

The excellent response (ER) rate for low- and intermediate-risk were 93.7% and 78.2%, respectively (P < 0.05). There were significant differences in age, cumulative dose of [^131^I], and pretreatment stimulated-Tg (pre-Tg) levels between the low- and intermediate-risk groups (P < 0.05). There were significant differences in the cumulative dose of ^131^I and pre-Tg levels between ER and the non-ER group (P < 0.05). The area under the curve (AUC) values were 0.799 in the low-risk group, and 0.747 in the intermediate-risk group for the ROC curve by ER status of pre-Tg. The ER rate with RAI treatment decreased with an increase in pre-Tg levels.

**Conclusion:**

Pre-Tg was an important factor for RAI treatment decision-making and prognostic evaluation and differed between low-risk and intermediate-risk DTC. Aggressive RAI therapy was recommended for low-risk DTC with pre-Tg ≥ 20.0 ng/mL and in intermediate-risk group with pre-Tg ≥ 10.0 ng/mL.

## INTRODUCTION

Thyroid cancer is one of the most common endocrine malignancies with a growing incidence worldwide ( 1 , 2 ). Papillary thyroid carcinoma (PTC) and follicular thyroid carcinoma (FTC), collectively referred to as differentiated thyroid cancer (DTC), account for more than 90% of all thyroid cancers. The conventional treatment options for DTC include surgical resection, postoperative selective radioactive iodine (RAI) therapy, and thyroid stimulating hormone (TSH) suppression therapy. Patients with intermediate- and high-risk DTC can benefit from postoperative RAI treatment, however, the 2015 American Thyroid Association (ATA) Guidelines and the domestic guidelines do not recommend RAI remnant ablation for low-risk DTC patients ( 3 , 4 ). Although, RAI treatment for low-risk DTC is not routinely recommended, a case-by-case recommendation of RAI treatment is frequently adopted among different nuclear medicine centers ( 5 ). Using the ATA risk stratification guidelines, patients are classified as being at low (<5%) or intermediate (5%-20%) risk of recurrence ( 3 , 6 ). Another study showed that nearly 10.3% of patients experience a recurrence in low-risk DTC and the recurrence rate increases during the prolonged follow-up ( 7 ). Potential benefits of RAI administration in low- and intermediate-risk DTC are still controversial. Studies have shown conflicting results in terms of recurrence in low-risk DTC patients treated with postoperative ^131^I ( 8 , 9 ). The choice of the therapeutic dose is more individualized for patients with low- and intermediate-risk DTC. In addition, there is no optimal RAI activity for remnant ablation, and low-dose RAI can achieve the same effect with fewer side effects ( 10 , 11 ). The results of studies testing these treatments vary between studies because of different inclusion criteria and varying conditions of the patients, for example. Our study utilized data from 423 patients with low- and intermediate-risk DTC who had been treated and followed. RAI treatment and influencing factors have been discussed to help provide reference for RAI treatment and selection for low- and intermediate-risk DTC patients.

## PATIENTS AND METHODS

### Subjects

A retrospective analysis of 423 low- and intermediate-risk DTC patients admitted to the Department of Nuclear Medicine, Sichuan Provincial People’s Hospital from January 2005 to December 2020 was performed. All DTC patients were treated with surgery, had a postoperative pathological diagnosis, and ^131^I treatment. There were 418 cases of papillary carcinoma, five cases of follicular carcinoma, 89 males, and 334 females. The average age was 41.0 ± 13.6 years. The duration of follow-up was 6.0-188 months, with a median follow-up of 72.0 months (interquartile range: 51.0-92.0 months).

The recurrence risk of DTC patients was stratified according to the 2015 ATA guidelines.

Patients with high-risk DTC, incomplete follow-up data, and lack of clinical information were excluded. Finally, 423 eligible DTC patients were enrolled in this study.

The patients received a second ablation with oral ^131^I 6 months following the first ablation. If the second ^131^I treatment failed again, the patients received a third treatment of ^131^I. The decision to repeat ^131^I administration was based on non-stimulated serum thyroglobulin (Tg) levels > 1.0 ng/mL or on abnormal uptake in the ^131^I whole-body scans (WBS). Repetitive ^131^I treatments were administrated until successful ablation was achieved, or until the patient was unable to tolerate or refused the treatment. Annual check-ups were performed along with measurements of non-stimulated Tg levels.

According to the Tumor, Node, Metastasis (TNM) staging criteria of the American Joint Cancer Council ( 12 ), there were 173 T1a staged cases, 93 T1b staged cases, 105 T2 staged cases, and 52 T3 staged cases. There were Nx9 cases (x indicates uncertain), 148 N0 cases; 111 Nla cases, and 155 Nlb cases. In the recurrence risk stratification, 143 cases were considered low-risk and 280 cases were considered intermediate-risk ( Table 1 ).

**Table 1:** Comparison of treatment frequency and therapy response between the low- and intermediate-risk DTC groups

	Risk stratification	Low risk	Intermediate-risk	F	P
Therapy response	ER	134 (93.7%)	219 (78.2%)	16.45	0.000
	IR	1	17		
	BIR	7	37		
	SIR	1	7		
Frequency of RAI	1 dose	129 (90.2%)	188 (67.1%)		
	2 doses	12 (8.4%)	68 (24.3%)		
	≥3 doses	2 (1.4%)	24 (8.6%)		
	Total	143 (100.0%)	280 (100.0%)		
ER% in different Pre-Tg (ng/mL) groups	≤1.0	39/40 (97.5%)	55/62 (88.7%)	85.021	0.000
	1.0~10	64/66 (97.0%)	87/96 (90.6%)		
	10~20	9/9 (100.0%)	29/37 (78.4%)		
	>20	5/10 (50.0%)	15/39 (38.5%)		

Chi-squared test; ER: excellent response; IR: indeterminate response: BIR: biochemical incomplete response; SIR: Structural incomplete response; Pre-Tg: pretreatment stimulated-Tg.

### Methods

Patients stopped using levothyroxine(L-T4) for 3-4 weeks prior to treatment and avoided using iodine-rich drugs and foods ( 3 ). Free triiodothyronine (FT3), free thyroxine (FT4), thyroid-stimulating hormone (TSH), pretreatment stimulated-Tg (pre-Tg), and thyroglobulin antibody (TgAb) levels were measured. Thyroid 24-hour radioiodine uptake (RAIU), chest X-ray or chest computed tomography (CT), neck ultrasound were performed. FT3, FT4, TSH, Tg and TgAb were determined by electrochemiluminescence using an automatic E170 analyzer (Roche, USA). The therapeutic dose of the first ^131^I treatment was 2.22-3.70 GBq, and that of cervical lymph node metastases was 5.55GBq.

^131^I-WBS was performed using single-photon emission computed tomography SPECT (Siemens, Symbia T2) 3-5 days after ^131^I administration. SPECT/CT fusion imaging was performed when residual thyroid or metastases were unable to be identified. Patients were instructed to resume L-T4 therapy 48 hours after administration of ^131^I. The results of whole-body imaging and tomographic fusion imaging were read and interpreted by two senior nuclear medicine doctors.

Efficacy evaluation and follow-up were performed according to the 2015 ATA guidelines, and were based on examinations, biomarker measurements (Tg, TgAb) and imaging (neck ultrasound, ^131^I whole-body imaging, chest CT). Efficacy of treatment was divided into four categories: satisfactory (excellent response, ER), indeterminate response (IR), biochemical incomplete response (BIR), and poor imaging response (structural incomplete response, SIR). All 423 DTC patients in this group were followed for 6-188 months, with a median follow-up of 72 months.

This study was conducted according to the guidelines laid down in the Declaration of Helsinki and all procedures involving research study participants were approved by the Ethics Committee of Sichuan Provincial People’s Hospital, Sichuan Academy of Medical Sciences.

### Statistical analysis

All statistical analyses were performed using the SPSS statistical software version 20.0 (SPSS Inc.). Analysis of variance was used to compare data between different groups. Data that was not normally distributed were represented by the median (with lower and upper quartiles). A Mann-Whitney U test and Kruskal-Wallis rank sum test were used for comparison between different groups. P < 0.05 was considered statistically significant. A chi-squared test was used to compare groups of ER and NER.

## RESULTS

Among the 143 cases with low-risk DTC, 129 patients were treated with one dose of RAI, 12 patients were treated twice with RAI, and three patients were treated at least three times with RAI. There were 280 patients with intermediate-risk DTC. Of these 280 patients, 188 patients were treated with one dose of RAI, 68 patients were treated twice with RAI, and 24 patients were treated at least three times with RAI. The ER rates for low- and intermediate-risk DTC were 93.7% (134/143) and 78.2% (219/280), respectively (P < 0.05). Pre-Tg levels were significantly correlated with ER in the low- and intermediate risk DTC groups (P < 0.05) ( Table 1 ).

There were significant differences in age, cumulative dose of ^131^I, frequency of doses of ^131^I, and pre-Tg levels between the low- and intermediate-risk groups (P < 0.05). However, there was no difference in 24-hour RAIU, FT3, FT4, TSH, and TgAb levels between the two groups (P > 0.05) ( Table 2 ).

**Table 2:** Comparison of clinical features in low- and intermediate-risk DTC

	Low risk	Intermediate-risk	Total	F	P
N	143	280	423
Age (y)	44.5 ± 13.1	39.2 ± 13.4	41.0 ± 13.6	3.899	0.000 a
Frequency of RAI	1.11 ± 0.36	1.45 ± 0.78	1.34 ± 0.69	6.146	0.000 a
cumulative dose ^131^I(mci)	113.1 ± 46.1	162.4 ± 117.6	145.8 ± 102.0	6.147	0.000 a
24 h RAIU(%)	2.9 (1.7,7.9)	3.5 (1.8,6.4)	3.3 (1.7, 6.9)	0.548	0.459 a
FT3 (pmol/L)	2.35 ± 0.89	2.30 ± 0.90	2.32 ± 0.89	0.480	0.631 a
FT4 (pmol/L)	5.83 ± 1.94	5.87 ± 2.16	5.86 ± 2.09	0.186	0.852 a
TSH (mIU/L)	79.8 ±2 8.7	81.9 ± 27.6	81.2 ± 28.0	0.712	0.477 a
Tgab (IU/mL)	10.3 (6.13, 25.1)	12.3 (5.0, 22.92)	11.4 (5.2, 23.6)	0.411	0.681 b
Pre-Tg (ng/mL)	2.46 (0.49, 6.78)	3.92 (0.89, 14.2)	3.42 (0.71, 10.6)	2.909	0.004 b
Follow-up (months)	72.7 ± 30.0	69.6 ± 32.6	70.6 ± 31.7	0.942	0.347 a

a Independent-sample *t* -test.

b Tg. TgAb: nonparametric rank sum test, Mann-Whitney U test. Pre-Tg: pretreatment stimulated-Tg.

Age, number of treatments, cumulative dose, and pre-Tg levels were significantly different between different Nodal stages (P < 0.05). However, there was no significant difference in 24-hour RAIU, FT3, FT4, TSH, and TgAb levels (P > 0.05). There was significant difference in age, doses of RAI treatment, cumulative dose of ^131^I, pre-Tg levels, 24-hour RAIU, FT3, FT4, and TSH levels among different Tumor stages (P < 0.05). However, there was no significant difference in TgAb levels observed ( Table 3 ).

**Table 3:** Comparison of parameters for different tumor and nodal stages

	N stage	N	Mean ± SD	F	P	T stage	N	Mean ± SD	Z	P
Age (year)	N0+Nx	151	44.4 ± 13.1	9.328	0.000	T1	266	41.8 ± 13.2	6.229	0.002
	N1a	111	40.8 ± 13.0			T2	105	37.3 ± 13.6		
	N1b	161	37.9 ± 13.6			T3	52	44.4 ± 13.8		
Frequency of RAI	N0+Nx	151	1.1 ± 0.4	29.828	0.000	T1	266	1.2 ± 0.4	21.59	0.000
	N1a	111	1.2 ± 0.4			T2	105	1.6 ± 0.9		
	N1b	161	1.6 ± 0.9			T3	52	1.6 ± 0.9		
Cumulative dose ^131^I (mci)	N0+Nx	151	113.8 ± 47.5	28.99	0.000	T1	266	122.2 ± 59.2	20.96	0.000
	N1a	111	124.1 ± 57.7			T2	105	183.8 ± 138.4		
	N1b	161	190.7 ± 140.4			T3	52	189.4 ± 145.0		
24h RAIU (%)	N0+Nx	151	5.5 ± 5.8	0.87	0.420	T1	265	4.8 ± 5.2	5.226	0.006
	N1a	111	5.0 ± 5.8			T2	104	6.6 ± 6.9		
	N1b	159	6.0 ± 6.2			T3	52	7.1 ± 6.8		
TSH (mIU/L)	N0+Nx	151	79.7 ± 29.3	0.329	0.720	T1	266	83.3 ± 24.7	3.271	0.039
	N1a	111	82.2 ± 23.5			T2	105	80.1 ± 30.8		
	N1b	161	81.9 ± 29.7			T3	52	72.7 ± 35.9		
FT3 (pmol/L)	N0+Nx	151	2.4 ± 1.0	2.083	0.126	T1	266	2.20 ± 0.73	8.712	0.000
	N1a	111	2.20 ± 0.67			T2	105	2.45 ± 0.96		
	N1b	160	2.37 ± 0.95			T3	51	2.70 ± 1.31		
FT4 (pmol/L)	N0+Nx	151	5.91 ± 2.12	0.789	0.455	T1	266	5.59 ± 1.47	6.306	0.002
	N1a	111	5.65 ± 1.65			T2	105	6.27 ± 2.79		
	N1b	160	5.96 ± 2.32			T3	51	6.43 ± 2.82		
Tgab (IU/mL)	N0+Nx	137	10.27 (5.63,25.11)	3.942	0.139	T1	225	12.75 (5.95,25.6)	5.961	0.051
	N1a	100	13.27 (5.81,28.60)			T2	90	9.40 (4.60,21.95)		
	N1b	122	10.73 (4.88,19.78)			T3	44	8.50 (2.52,16.51)		
Pre-Tg (ng/mL)	N0+Nx	137	2.55 (0.46,7.45)	20.707	0.000	T1	225	2.21 (0.53,6.70)	27.713	0.000
	N1a	100	2.36 (0.78,6.85)			T2	90	6.78 (1.03,19.78)		
	N1b	122	7.34 (1.13,22.05)			T3	44	9.63 (2.96,19.53)		

ANOVA; Tg, TgAb: nonparametric rank sum test, Kruskal-Wallis rank sum test. X: indicated uncertain.

There was significant difference in cumulative dose of ^131^I, and FT3, FT4, and TgAb levels among various pre-Tg level groups (P < 0.05) ( Table 4 ).

**Table 4:** Comparison of parameters in different pre-Tg level groups

	Pre-Tg (ng/mL)	N	Mean	F	P
Pre-Tg (ng/mL)	≤1.0	102	0.2 (0.20, 0.545)	316.194	0.000
	1.0~10	162	3.51 (2.01, 5.78)		
	10~20	46	13.7 (11.48, 15.73)		
	>20	49	35.60 (24.50, 84.75)		
Frequency of RAI	≤1.0	102	1.1 ± 0.4	15.114	0.000
	1.0~10	162	1.2 ± 0.5		
	10~20	46	1.4 ± 0.6		
	>20	49	2.1 ± 1.0		
Cumulative dose (mci)	≤1.0	102	116.2 ± 46.8	14.287	0.000
	1.0~10	162	125.8 ± 79.5		
	10~20	46	147.8 ± 69.1		
	>20	49	263.3 ± 164.2		
24h RAIU (%)	≤1.0	102	2.3 ± 1.7	37.633	0.000
	1.0~10	162	4.9 ± 4.7		
	10~20	46	10.7 ± 8.0		
	>20	49	8.8 ± 7.2		
Tgab (IU/mL)	≤1.0	102	25.0 (12.25, 50.46)	64.776	0.000
	1.0~10	162	9.60 (4.98, 18.54)		
	10~20	46	7.89 (3.23, 14.88)		
	>20	49	7.80 (2.50, 13.22)		

ANOVA; Tg, TgAb: Kruskal-Wallis rank sum test. Pre-Tg: pretreatment stimulated-Tg.

There was significant difference in cumulative dose of ^131^I and pre-Tg levels between ER and NER groups (P < 0.05) ( Table 5 ).

**Table 5:** Comparison of parameters between ER and NER groups

	ER	N	NER	N	F	P
Age (year)	41.1 ± 13.0	353	40.6 ± 14.7	70	0.243	0.808 a
^131^I cumulative dose (mci)	125.4 ± 70.7	353	248.6 ± 159.0	70	10.319	0.000 a
24h RAIU (%)	5.34 ± 5.79	353	6.52 ± 6.53	70	1.524	0.128 a
FT3 (pmol/L)	2.28 ± 0.86	353	2.51 ± 1.04	70	1.991	>0.047 a
FT4 (pmol/L)	5.78 ± 1.95	353	6.19 ± 2.65	70	1.190	0.237 a
TSH (mIU/L)	81.9 ± 27.1	353	77.9 ± 32.3	70	1.094	0.275 a
Tgab (IU/mL)	13.3 (6.6,39.7)	303	15.1 (5.81,46.1)	56	1.725	0.085 b
Pre-Tg (ng/mL)	1.73 (0.34,5.66)	303	2.61 (0.42,11.6)	56	6.295	0.000 b

a Independent-sample t-test.

b Tg. TgAb: nonparametric rank sum test, Mann-Whitney U test. Pre-Tg: pretreatment stimulated-Tg.

**Figure 1:**
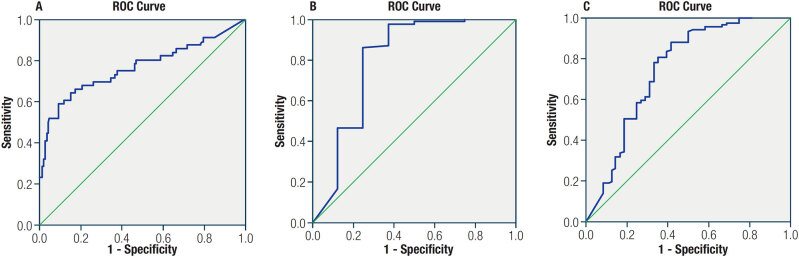
( **A** ) ROC curve for ER by pre-Tg status in low- and intermediate-risk groups (AUC = 0.764). ( **B** ) ROC curve for ER by pre-Tg status in the low-risk group (AUC = 0.799). ( **C** ) ROC curve for ER by pre-Tg status in then intermediate-risk group (AUC = 0.747).

ROC curves for the relationship between pre-Tg levels and ER were established. The area under the curve (AUC) values were 0.764 ( Figure 1A ) in the whole group, 0.799 in the low-risk group ( Figure 1B ), and 0.747 in the intermediate-risk group ( Figure 1C ). With pre-Tg levels of 10.0 ng/mL as the cutoff, the sensitivity and specificity were 88.0% and 62.5% in the low-risk group, respectively, and 75.8% and 66.7% in the intermediate-risk group, respectively. With pre-Tg levels of 15.6 ng/mL as the cutoff, the sensitivity was 88.2% and the specificity was 58.3% in the intermediate-risk group.

## DISCUSSION

RAI treatment is used in DTC patients to remove residual thyroid lesions, as adjuvant therapy, and for the treatment of metastases. RAI treatment after total thyroidectomy in DTC patients is beneficial to prolong disease-specific survival, reduce recurrence, help initial staging and to improve detection during long-term follow-up ( 3 ). The peri-RAI findings are essential in order to carefully stratify the recurrence risk of DTC patients. Patients who are reclassified as being at high-risk after RAI have a long-term follow-up similar to patients at high-risk just post-surgery ( 13 ). The factors determining postoperative RAI administration should be based on risk factor assessment, recurrence risk stratification, TNM stage, the quality of surgery, serum Tg, adverse reactions from RAI, doctor treatment recommendations, and patient wishes ( 14 ).

Postoperative risk stratification can guide the decision for RAI treatment and correlates with clinical outcome with RAI therapy. Risk stratification is also significantly related to the frequency of treatments and cumulative dose of RAI. More than 80% of DTC patients are currently classified as being at low risk with less than < 5% of recurrence in ATA risk stratification ( 3 ). Our study showed that ER rates from RAI treatment in low-risk and intermediate-risk DTC patients were 93.7% and 78.2%,respectively. However, nearly 7.0% of low-risk DTC patients did not achieve complete remission. Being able to distinguish the potential recurrence risk of low-risk DTC patients would be helpful for RAI treatment selection and follow-up.

TNM staging and risk stratification are used as prognostic classifications of DTC.TNM stage is related to disease-specific death risk. A study by Cao and cols. that included 357 DTC patients with cervical lymph node metastases revealed that tumor size, number of nodules, and TNM stage were independent risk factors associated with successful ablation in DTC patients who received two doses of RAI ( 15 ). Our study showed that TN stages were significantly related to age, frequency of RAI, cumulative dose, and pre-Tg levels. Our data indicated that TN stage helped stratify recurrence risk, as well as identify treatment options and outcomes. One of the factors contributing to the variability seen in ER from various published studies could be the differences in TN stage to the DTC patients ( 11 ). Age, RAI dose, risk stratification, size of thyroid remnants, and Tg levels are predictors influencing therapeutic response to RAI treatment ( 16 - 19 ).

Therapeutic dose is another factor affecting therapy response, where the amount of radioactivity from RAI is aimed to be as low as feasible to avoid unnecessary radiation exposure in low- and intermediate-risk DTC patients. Options for RAI treatment and dosing in low- and intermediate-risk DTC patients vary significantly among different centers. Some studies have reported that low-dose (1.1 GBq) RAI has the same effect as medium- and high-dose (3.7GBq) RAI ( 20 ). However, other studies have given opposite conclusions ( 21 ). Albano and cols. found that the ablation rate with intermediate-high RAI activity (1.85-3.7 GBq) was better than with low (1.1 GBq) RAI activity. WBS may help to identify nodal and distant metastases in about 10% of cases, thereby altering the clinical stage and subsequent management ( 21 ). Ma and cols. analyzed a cohort of Chinese DTC patients and showed that a low dose of 1,850 MBq radioiodine was as effective as a high dose of 3,700 MBq for thyroid remnant ablation ( 22 ). Our study used 3.7 GBq to ablate remnant thyroid lesions with an overall ER rate of 83.5%, lower than the rate reported by Dong and cols. ( 11 ). The difference in RAI remnant thyroid ablation observed among studies may be because of differences in inclusion criteria, TNM stage, and pre-Tg levels, etc.

Our study showed that there was no significant difference in FT3 and FT4 levels and ^131^I uptake between the low- and intermediate-risk groups, but there was a significant difference in pre-Tg levels. Significant differences in pre-Tg levels were found between the ER group and non-ER group. pre-Tg was the most important parameter in the choice of RAI therapy and closely related to the therapeutic response. Tg levels play a key role during long-term follow-up of DTC patients ( 23 , 24 ). In patients undergoing total thyroidectomy and RAI treatment, there is no remaining normal thyroid tissue in the body, resulting in undetectable Tg levels. Patients with intermediate-risk DTC and undetectable postoperative serum Tg levels can be considered as having a low risk of recurrence ( 25 ). A high postoperative Tg level indicates more thyroid remnants and lesions exist. However, there was not a well-established cutoff for serum Tg levels to differentiate the source of Tg, whether from benign thyroid remnants, occult tumor tissue, or even structural persistent disease ( 26 ). Mousa and cols. showed that in DTC patients negative for TgAb, ablative stimulated Tg levels ≥ 5.6 ng/mL was identified as a cutoff correlating with increased risk of recurrence by 2.38-fold, whereas an late stimulated Tg obtained 6-12 months after primary therapy ≥ 0.285 ng/mL increased the risk of relapse by 3.087-fold ( 27 ). Ben Ghachem and cols. demonstrated that a baseline Tg level ≥ 10 ng/mL was a significant independent predictor of successful first cure ablation ( 28 ). A study by Ha and cols. indicated that pre-ablative Tg levels was the only significant factor related with ablation success rates. Pre-ablative Tg levels with a cutoff value of 10.0 ng/mL is a promising factor to predict successful remnant ablation ( 29 ). Abe and cols. showed that Tg levels were the only independent powerful predictive factor for successful RAI ablation. The best cutoff value of Tg levels for predicting unsuccessful ablation was 9 ng/mL. Low-dose RAI routinely performed in Japan may be inadequate for the achievement of successful ablation. For patients with Tg levels > 9 ng/mL at the time of the first RAI therapy, a higher dose of RAI is recommended ( 30 ). In pediatric DTC patients, postoperative high stimulated Tg values (>10 ng/mL) should be taken into account for deciding the extent of both initial treatment and follow-up ( 31 ). The above published studies have shown that the cutoff of pre-stimulated Tg levels ≥ 10.0 ng/mL may be a threshold for prediction of therapy response. Our study indicated pre-Tg levels of 10.0 ng/mL as the cutoff for ER. The sensitivity and specificity were 88.0% and 62.5% for the low-risk group, respectively, and 75.8% and 66.7% for the intermediate-risk group, respectively. Zakavi and cols. indicated that Tg levels were the most potent risk factor for prediction of treatment failure and Tg levels ≥ 35.5 ng/mL were the most important factor for prediction of incomplete response ( 32 ). Tg cutoff levels differed in various publications during diagnosis and follow-up. However, there was no comparison for the same level among different risk stratification groups. Our data indicated that the ER rate gradually decreased with pre-Tg level increases and the pre-Tg cutoff value differed in different DTC risk stratification groups. The ER rate was significantly reduced in low-risk DTC with an ER of 50.0% in the pre-Tg > 20.0 ng/mL group, while the ER rate was 78.4% in intermediate-risk group with pre-Tg 10-20.0 ng/mL, and RAI treatment is recommended to this group. Yang and cols. found that patients with more advanced disease at the initial risk stratification level were more likely to have higher pre-Tg levels. The median Tg levels for the low-, intermediate-risk and high-risk groups were 1.7, 4.4 and 14.7 ng/mL, respectively ( 33 ).

The interference of TgAb must be considered when Tg levels are assessed. Serum TgAb were found in 10%-30% of DTC patients, and could interfere in the Tg detection assays leading to false-negative results. The change in TgAb titers over time can be used as an alternative tumor biomarker ( 34 , 35 ). In this article, we found that there was no difference in therapy response from RAI between patients positive for TgAb (TgAb+) and negative for TgAb (TgAb-). The data indicated RAI treatment was equally effective both in TgAb+ and TgAb- groups. However, pre-Tg levels in the TgAb+ group were significantly lower than those in the TgAb- group, suggesting that the value of Tg is limited in the TgAb+ group. There are numerous methods for the clinical detection of serum Tg levels, such as radioimmunoassay, and chemiluminescent immunoassay. Tg level test results vary widely because of measurement at different laboratories, with different instruments, reagents and test methods, so results are incomparable. In our study, the detection of Tg and TgAb was performed in the same laboratories and used the instruments. Therefore, the results were comparable.

There were a few limitations in the present study. This was a retrospective study, the sample size was small, and the study was at a single center. The results will require a prospective confirmation in future studies. Side effects of RAI treatment, such as transient radiation, thyroiditis, head and neck discomfort, dysfunction of the digestive system, salivary glands, and gonads have not been analyzed. In addition, most of the patients had a long-term follow-up with a median follow-up of 72 months. However, a few patients were not followed for long periods.

In conclusion, the present study included the long-term follow-up of 423 DTC patients at low- and intermediate-risk, with an ER rate of 83.5% at a median follow-up of 72 months. Pretreatment stimulated-Tg (pre-Tg) levels in DTC patients was an important indicator in RAI treatment decision-making and efficacy evaluation. DTC patients would benefit from aggressive RAI therapy in the low risk group with pre-Tg ≥ 20.0 ng/mL and in intermediate-risk group with pre-Tg ≥ 10.0 ng/mL.
